# Case Report: An Unusual Case of Biventricular Thrombosis in a COVID-19 Patient With Ischemic Dilated Cardiomyopathy: Assessment of Mass Mobility and Embolic Risk by Tissue Doppler Imaging

**DOI:** 10.3389/fcvm.2021.694542

**Published:** 2021-07-29

**Authors:** Andrea Sonaglioni, Adriana Albini, Gian Luigi Nicolosi, Elisabetta Rigamonti, Douglas M. Noonan, Michele Lombardo

**Affiliations:** ^1^Department of Cardiology, Istituto di Ricovero e Cura a Carattere Scientifico (IRCCS) Multi Medica, Milan, Italy; ^2^Scientific and Technological Pole, Istituto di Ricovero e Cura a Carattere Scientifico (IRCCS) Multi Medica, Milan, Italy; ^3^Department of Cardiology, Policlinico San Giorgio, Pordenone, Italy; ^4^Department of Biotechnology and Life Sciences, University of Insubria, Varese, Italy

**Keywords:** COVID-19, biventricular thrombosis, pulsed wave tissue Doppler imaging, ACE2, dilated cardiomyopathy

## Abstract

Severe acute respiratory syndrome coronavirus 2 (SARS-CoV-2) spike protein binds to angiotensin-converting enzyme 2 (ACE2) receptor on vascular cells. As a consequence, patients with COVID-19 have an increased incidence of thromboembolic complications of the SARS-CoV-2 infection and subsequent endothelial cell damage with consequence of development of systemic vasculitis and diffuse intravascular coagulation. The present case describes a COVID-19 female patient with ischemic dilated cardiomyopathy, who presented with congestive heart failure and echocardiographic evidence of biventricular apical thrombi. The peak antegrade longitudinal velocity (Va) of each thrombotic mass was measured by pulsed wave tissue Doppler imaging (PW-TDI). Both left ventricular and right ventricular apical thrombi were found with a TDI-derived mass peak Va < 10 cm/s. There was no clinical evidence of neither systemic nor pulmonary embolization, probably due to the hypomobility of both left and right ventricular masses.

## Introduction

Left ventricular (LV) thrombosis can complicate both ischemic and non-ischemic cardiomyopathies and can lead to arterial embolic complications such as stroke (1, 2). However, the occurrence of biventricular thrombi is very rare and only few cases have been previously described in literature (3–9). Here, we report the case of an 80-year-old woman infected by Coronavirus 2019 (COVID-19), presenting with congestive heart failure (CHF) due to ischemic dilated cardiomyopathy (DCM), who was diagnosed with biventricular apical thrombi by transthoracic echocardiography (TTE).

## Clinical Course

An 80-year-old woman, BSA 1.62 m^2^, body mass index (BMI) 22.6 Kg/m^2^ with history of coronary artery disease presented to the Emergency Department (ED) with worsening dyspnea, non-productive cough, fatigue, and bilateral leg swelling. She had prior anterior myocardial infarction treated with percutaneous transluminal coronary angioplasty of the proximal left anterior descending coronary artery in 2019 and subsequent unfavorable evolution in DCM with severe systolic dysfunction (estimated left ventricular ejection fraction of 20%) and chronic renal failure (estimated glomerular filtration rate of 30 ml/min/1.73 m^2^). She was in home-therapy with acetyl salicylic acid 75 mg/die, furosemide 50 mg/die, spironolactone 25 mg/die, and rosuvastatin 5 mg/die.

Parameters recorded at the admission were the following: body temperature 36.5°C, heart rate 82 beats per minute, blood pressure 150/90 mmHg, respiratory rate 28 times per minute, and oxygen saturation 90% on ambient air.

Blood tests showed a white blood cell count of 8,720/mmc (88.0% neutrophils and 8.0% lymphocytes), hemoglobin 16.5 g/dl, C-reactive protein at the level of 11.1 mg/dl (reference range 0.05–0.50 mg/dl), estimated glomerular filtration rate 25 ml/min/1.73 m^2^, B-type natriuretic peptide level >20,000 pg/ml, D-dimer at the level of 17,108 ng/ml (reference range 1–500 ng/ml), and troponin I at the level of 0.08 ng/ml (reference range 0.00–0.04 ng/ml).

The electrocardiogram showed sinus rhythm, poor R wave progression in precordials, suggestive of old large anterior myocardial infarction and QRS voltage <5 mm in all limb leads.

**Figure 1:**
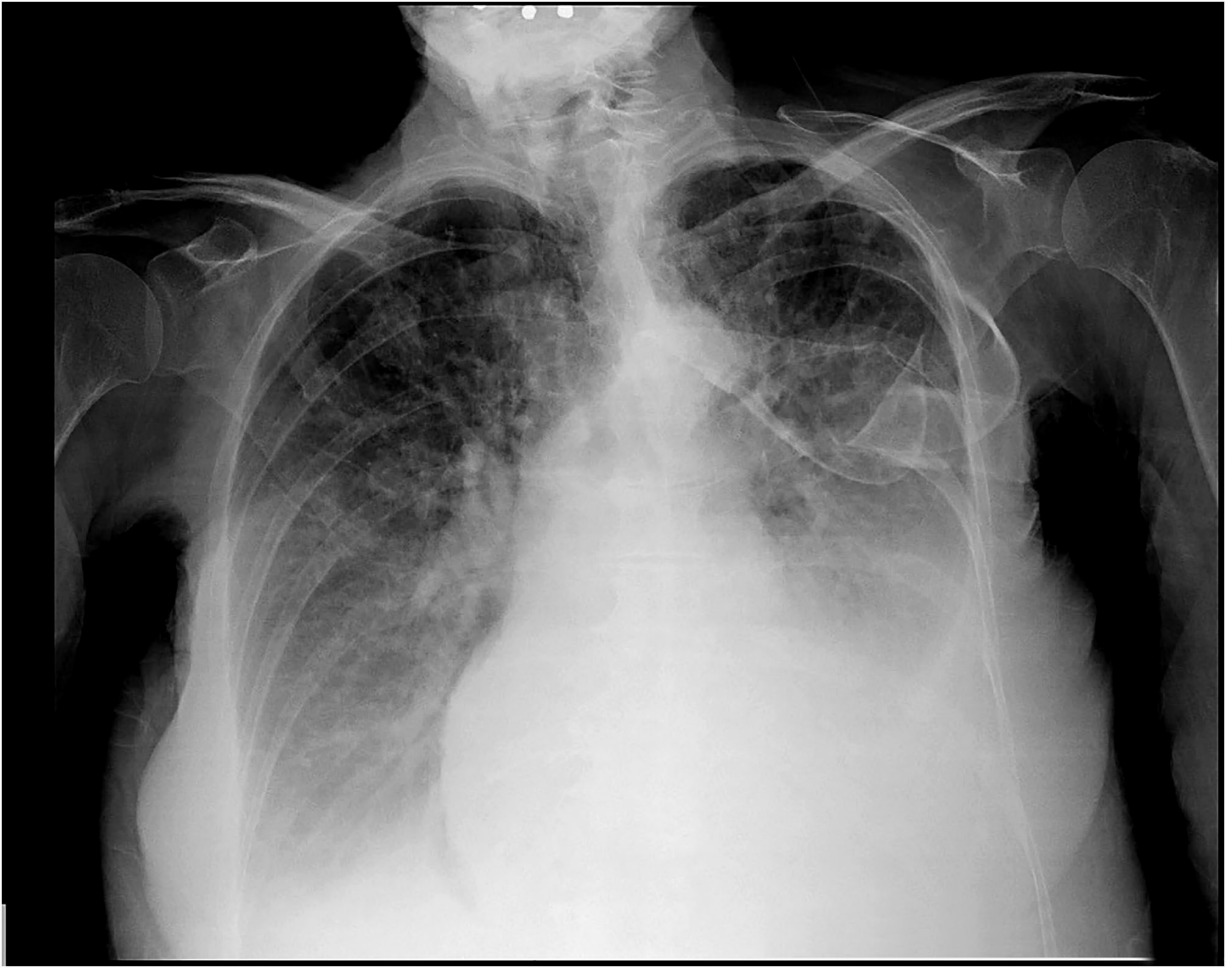
Posteroanterior chest x-ray view revealing cardiomegaly, bilateral interstitial infiltrates and bilateral pleural effusions.

Chest x-ray showed cardiomegaly, bilateral interstitial infiltrates and bilateral pleural effusions (Figure 1). A positive rapid antigen test for Severe Acute Respiratory Syndrome Coronavirus 2 (SARS-CoV2) was confirmed by the molecular test at hospital admission. Therefore, the patient was transferred from the ED to the semi-intensive care unit for COVID-19 patients.

A bedside TTE revealed the presence of large and roundish masses in the apex of both ventricles associated with severe biventricular dilatation (LV end-diastolic short axis diameter of 65 mm, right ventricular inflow tract of 45 mm) and dysfunction. Left ventricular ejection fraction (LVEF) estimated by modified Simpson's method was 15%, with dyskinesia/aneurism of the apex, and interventricular septum and marked hypokinesis of the other segments of the left ventricle. Right ventricular (RV) systolic function, measured by the tricuspid annular plane systolic excursion (TAPSE), was also severely impaired (TAPSE = 9 mm).

Both masses were acoustically distinct from underlying myocardium, with hypoechoic central space and hyperechoic border, well-circumscribed and sessile, attached to the apex of the left ventricle and of the right ventricle. The LV mass measured 23 mm × 21 mm, while the RV mass measured 18 mm × 15 mm. Both the masses were hypomobile and prominent in the apical 4-chamber view. Bilateral ventricular thrombi were diagnosed.

To precisely assess the mobility of the intracardiac thrombi, we employed pulsed-wave tissue Doppler imaging (PW-TDI) placing the sample volume at the level of the body of each mass. The peak antegrade longitudinal velocity (Va) of both LV and RV apical thrombi was measured.

**Figure 2:**
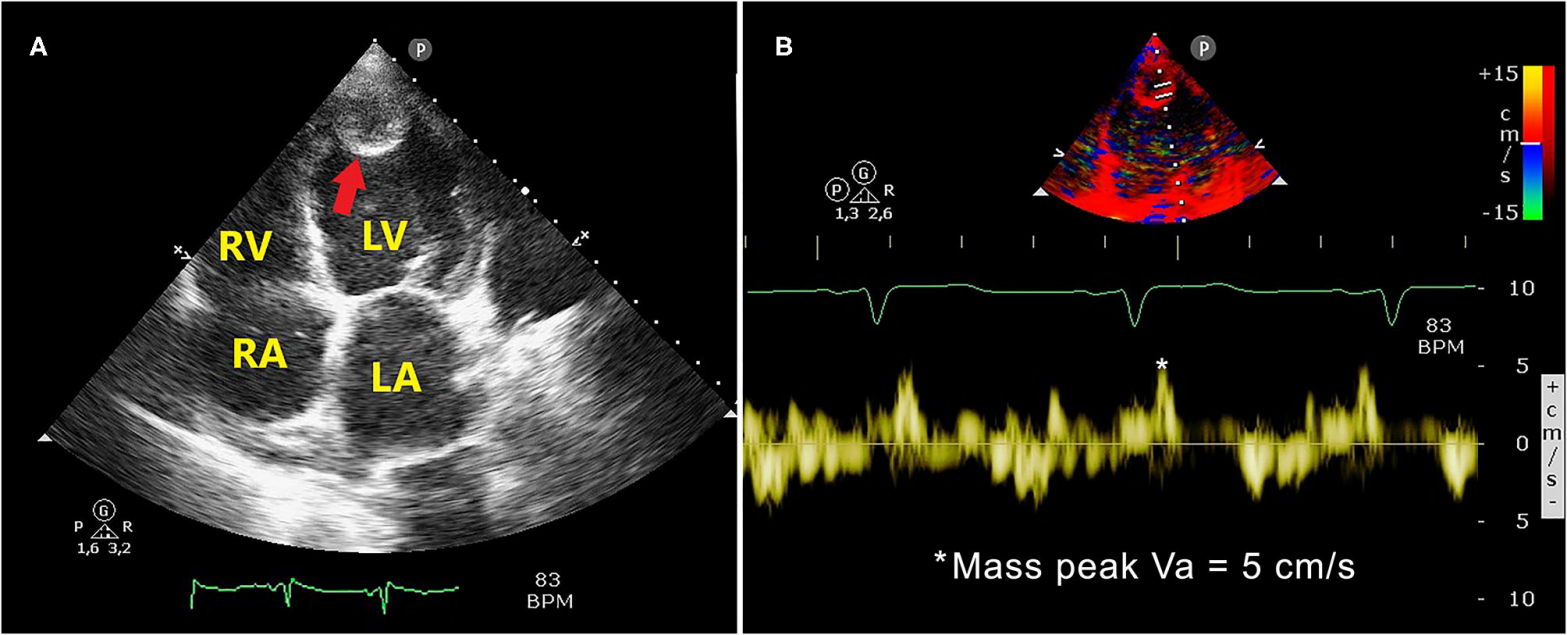
Apical 4-chamber echocardiographic view showing a large and roundish LV apical thrombus with hypoechoic central space and hyperechoic border (**A**, red arrow). The LV thrombotic mass measured 23 mm × 21 mm. The TDI-derived LV mass peak Va was 5 cm/s, as depicted in panel **(B)**. LA, left atrium; LV, left ventricular/left ventricle; TDI, tissue Doppler imaging; RA, right atrium; RV, right ventricle; Va, antegrade velocity.

**Figure 3:**
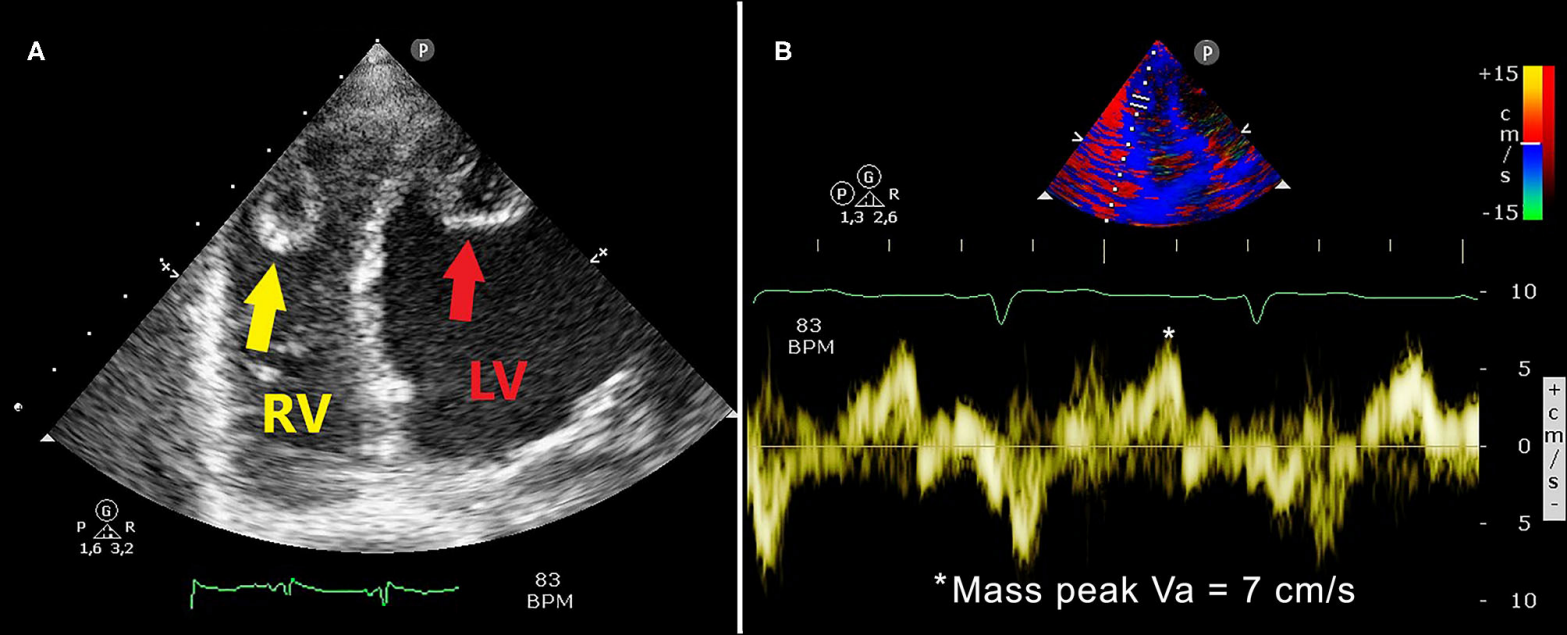
Right ventricular focused 4-chamber echocardiographic view demonstrating RV (**A**, yellow arrow) and LV (**A**, red arrow) apical thrombi: both masses were found with hypoechoic central space and hyperechoic border. The RV thrombotic mass measured 18 mm × 15 mm. The TDI-derived RV mass peak Va was 7 cm/s, as depicted in panel **(B)**. LV, left ventricular/left ventricle; TDI, tissue Doppler imaging; RV, right ventricular/right ventricle; Va, antegrade velocity.

Figure 2 illustrates the LV apical thrombus (Figure 2A) and the corresponding TDI-derived peak mass Va (Figure 2B), while Figure 3 depicts the RV apical thrombus (Figure 3A) and the relative TDI-derived peak mass Va (Figure 3B). Moderate mitral and tricuspid regurgitation, dilatation of the inferior vena cava and moderate pulmonary hypertension (the estimated systolic pulmonary artery pressure was 60 mmHg) were also detected.

The above-mentioned echocardiographic examination was compared with a previous TTE performed in October 2020 that showed severe biventricular dilatation and dysfunction (LV ejection fraction of 20%, TAPSE of 13 mm), with no evidence of ventricular thrombi.

The diagnosis of CHF due to ischemic DCM complicated with biventricular thrombi in a COVID-19 patient was made. Thrombosis at other sites was excluded; no deep vein thrombosis of the abdomen and lower extremities was found by ultrasonography.

Conventional treatment for CHF with loop diuretics (intravenous furosemide 120 mg/die and canrenone 100 mg/die) and beta-blockers (bisoprolol 2.5 mg/die) was started. Moreover, the patient received antibiotic treatment (intravenous piperacillin and tazobactam three times daily) and oxygen therapy via nasal cannula (2 l/min). Low molecular weight heparin (enoxaparin sodium) was administered for the treatment of biventricular thrombosis (4,000 IU twice daily by subcutaneous injection).

During the hospitalization, the patient underwent diagnostic thrombophilia testing. Results showed that serum levels of protein C, protein S, antithrombin III, factor V Leiden, and antiphospholipid antibody were normal.

**Figure 4:**
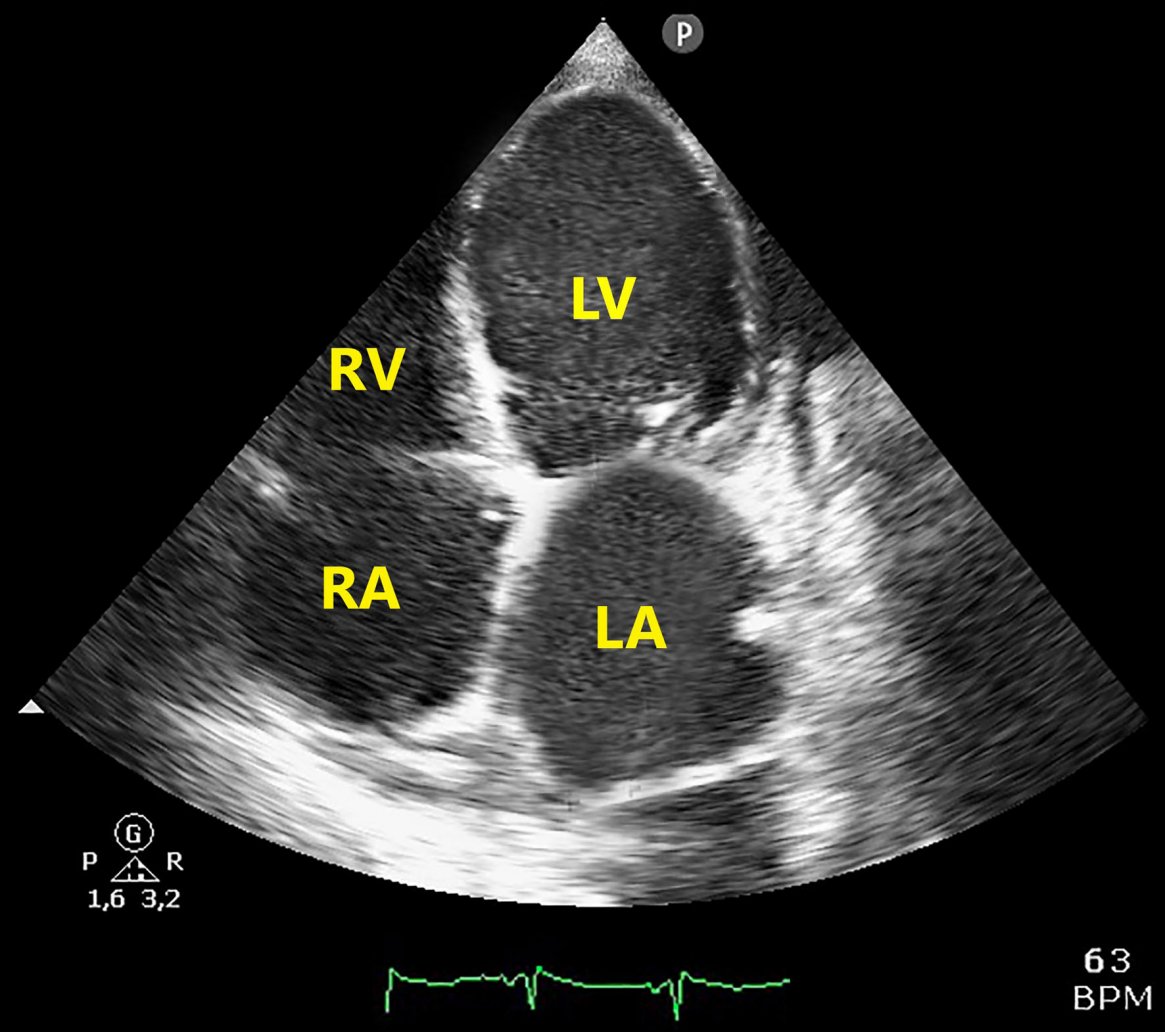
Apical 4-chamber echocardiographic view showing disappearance of both RV and LV thrombotic masses after 10 days of anticoagulant treatment. LA, left atrium; LV, left ventricle; RA, right atrium; RV, right ventricle.

A subsequent TTE, performed after 10 days of anticoagulant treatment, showed the complete dissolution of both left and right ventricular thrombi (Figure 4). There was no clinical evidence of neither systemic nor pulmonary embolization, probably due to the hypomobility of both left, and right ventricular masses.

However, severe biventricular dysfunction persisted, and refractory CHF occurred, despite intensive diuretic therapy. The patient underwent serial chest x-ray which showed progressive increase of pulmonary congestion and interstitial infiltrates. Computed tomography scan was not performed due to the critical condition of the patient and the absence of clinical signs of pulmonary/systemic embolization. Finally, the patient's clinical conditions worsened, and she died 15 days after hospitalization.

## Discussion

Severe acute respiratory syndrome coronavirus 2 (SARS-CoV-2) spike protein binds to angiotensin-converting enzyme 2 (ACE2) receptor on vascular cells (10, 11). On endothelial cells from arterial and venous vessels there is ACE2 expression and there is evidence that endothelial cells are prone to SARS-CoV-2 infection which causes subsequent endothelial cell damage with development of systemic vasculitis and disseminated intravascular coagulation (DIC) (10). Since the beginning of the COVID-19 pandemic, severe hypercoagulability and serious thrombotic complications have been reported in infected patients, especially in those patients who are admitted to intensive care unit (12–16). The most common thromboembolic complications detected in COVID-19 patients were deep vein thrombosis, acute pulmonary embolism, coronary and cerebral thrombosis, systemic arterial embolism, and placental thrombosis (17–21).

The occurrence of biventricular thrombi is a rare, but serious condition which may increase the risk of both systemic and pulmonary embolization. Previous cases of biventricular thrombosis have been described in patients with severe ventricular dysfunction, autoimmune disease, HIV infection, nephrotic syndrome, hypereosinophilic syndrome, heparin-induced thrombocytopenia, and antiphospholipid syndrome (3–9).

To date, there are only three case reports in literature who described biventricular thrombi in a COVID-19 patient (22–24).

One patient, a 63-year-old woman, with a known medical history of emphysema and active smoker, 2-week history of worsening dyspnea, nonproductive cough, and chills, she was positive for SARS-CoV-2 as detected by PCR (22). After, few hours from admission she had a cardiac arrest with successful cardiorespiratory resuscitation. The patient had 93% oxygen saturation, elevated troponin I, creatinine kinase, and lactate, with normal platelet count, coagulation parameters, and fibrinogen (22). Cardiac tomography revealed a right ventricular thrombus, measuring 4 mm by 10 mm, a left ventricular thrombus 12-mm in thickness extending over a 6-cm perimeter (22). The patient died of cardiogenic and pulmonary septic shock.

Another patient, a 58-year-old man, had obesity (BMI of 31 kg/m^2^ on admission) and hypertension, he presented with intermittent fever and worsening shortness of breath on exertion he was positive for PCR SARS-CoV-2 (23). The patient initial clinical examination was normal except for an outstanding oxygen requirement. The patient had high C-reactive protein (CRP) D-dimer was significantly elevated, with normal prothrombin time and activated partial thromboplastin time, no pulmonary thromboembolism appeared on CT pulmonary angiography (23). On day 4 the D-dimer levels were noted to have risen, and on day 9 they were steadily rising. On day 9, CT pulmonary angiography revealed simultaneous bilateral pulmonary thromboembolism, biventricular cardiac thrombi (23). The patient with multiple thromboses had appropriate prophylactic and therapeutic LMWH is this case (23). The patient was successfully discharged on day 19.

A 58-year-old African-American male, with a history of hypertension and diabetes mellitus, was brought to emergency room (24). He had 60% oxygen saturation the clinical laboratory test showed a hypercoagulable state (Fibrinogen was low and high D-dimer levels, PT 36 s, INR 3.6, and aPTT of 100 s) normal troponin I, CK, CK-MB at normal levels (24). He was taking care of parents, confirmed positive with COVID-19. In this patient transthoracic echocardiography has seen left ventricle extensive mural thrombus and highly mobile thrombus the in right atrium with extensive biventricular thrombi (24). The patient died of ventricular fibrillation within 24 h.

Obesity is one of the complications of the COVID-19 patients (25). The BMI of the African-American male, with a history of hypertension and diabetes mellitus, and 63-year-old woman active smoker and emphysema was not mentioned in these case reports (22, 24). In all the case reports of biventricular thrombi and COVID-19 the patients were ~60 years.

In this case report, a 80 years COVID-19 patient with CHF due to ischemic DCM was diagnosed with LV and RV apical thrombi. Our patient had severely depressed systolic biventricular function, suggesting a high risk of ventricular thrombi. However, she was never diagnosed with ventricular thrombi prior to admission and was tested negative for thrombophilia screening during hospitalization. As far as we know, this is the first case of biventricular thrombi described in a COVID-19 patient with ischemic dilated cardiomyopathy.

As proposed by Mehta et al. (26), the combination of a progressive dysregulated coagulative response to SARS-CoV-2 with consequent activation of a state of hypercoagulability and the blood stasis due to severe biventricular dilatation and contractile dysfunction might have contributed to the formation of LV and RV apical thrombi.

In the present case, the D-dimer level of the patient was 17,108 ng/ml, well-above reference values, and the patient was treated with low-molecular-weight heparin, while the surgical thrombectomy was not considered due to the patient's advanced age and the severe CHF. The 63 years woman patient coagulation parameters were normal, but the authors mention the D-dimer (22). In the two male cases D-dimers were highly elevated (23, 24).

In the present case, contrast echocardiography, which is particularly advantageous for detection of small or mural thrombi (27), was not performed to confirm the diagnosis of LV and RV apical thrombi, because both thrombotic masses were large in size and protuberant in shape. On the other hand, PW-TDI was useful to precisely assessing the mobility of the intracardiac thrombi. Therefore, the diagnosis of biventricular thrombosis was performed and confirmed by TTE, whereas PW-TDI provided a rapid characterization of mass-mobility. Differently from previous case reports which described biventricular thrombi detected by TTE in different clinical settings (3–9), our case is the only one that employed a TTE implemented with PW-TDI assessment of the thrombotic mass mobility in a COVID-19 patient.

Our previous prospective analysis performed on 72 patients with echocardiographically detected LV thrombi revealed that a TDI-derived mass peak Va ≥ 10 cm/s, was the most important and independent predictor of outcome at mid-term follow-up (28). Therefore, we demonstrated that the TDI-derived mass peak Va might represent a new objective marker of thrombotic mass motility and that a mass peak Va ≥ 10 cm/s might stratify the hospitalized patients with increased probability of embolic events in a mid-term follow-up, regardless of the mass dimension.

In this present case, both LV and RV apical thrombi were found with a mass peak Va < 10 cm/sec, as assessed by PW-TDI, and the clinical course was not clinically complicated by systemic nor pulmonary embolization. However, the patient's clinical conditions quickly worsened due to SARS-CoV-2 severe pneumonia.

## Conclusion

This is a rare case of ischemic dilated cardiomyopathy complicated with biventricular apical thrombi early detected by TTE in a patient that was infected by COVID-19. The present case demonstrates the clinical usefulness of TTE implemented with PW-TDI for detecting ventricular thrombi and for measuring the thrombotic mass mobility. Although a biventricular thrombosis is a rare COVID-19 complication, performing appropriate diagnostic tests could decrease COVID-19 mortality in patients with dilated cardiomyopathy.

## Data Availability Statement

The raw data supporting the conclusions of this article will be made available by the authors, without undue reservation.

## Ethics Statement

Ethical review and approval was not required for the study on human participants in accordance with the local legislation and institutional requirements. Written informed consent for participation was not required for this study in accordance with the national legislation and the institutional requirements. Written informed consent was obtained for the publication of any potentially identifiable images or data included in this article.

## Author Contributions

AS, ER, and ML performed the provided clinical procedures and collected the clinical data. AS, GN, ER, and ML analyzed the data and prepared the figures. AS, AA, DN, and ML discussed data and wrote the manuscript. All authors contributed to the article and approved the submitted version.

## Conflict of Interest

The authors declare that the research was conducted in the absence of any commercial or financial relationships that could be construed as a potential conflict of interest.

## Publisher's Note

All claims expressed in this article are solely those of the authors and do not necessarily represent those of their affiliated organizations, or those of the publisher, the editors and the reviewers. Any product that may be evaluated in this article, or claim that may be made by its manufacturer, is not guaranteed or endorsed by the publisher.
